# Recent Progress on Stability of Layered Double Hydroxide-Based Catalysts for Oxygen Evolution Reaction

**DOI:** 10.3390/nano14181533

**Published:** 2024-09-21

**Authors:** Lielie He, Yangen Zhou, Mengran Wang, Simin Li, Yanqing Lai

**Affiliations:** School of Metallurgy and Environment, National Energy Metal Resources and New Materials Key Laboratory, Hunan Provincial Key Laboratory of Nonferrous Value-Added Metallurgy, Central South University, Changsha 410083, China; 243506110@csu.edu.cn (L.H.); 222034@csu.edu.cn (M.W.); simin.li@csu.edu.cn (S.L.); laiyanqing@csu.edu.cn (Y.L.)

**Keywords:** green hydrogen, OER, LDHs, stability

## Abstract

Water electrolysis is regarded as one of the most viable technologies for the generation of green hydrogen. Nevertheless, the anodic oxygen evolution reaction (OER) constitutes a substantial obstacle to the large-scale deployment of this technology, due to the considerable overpotential resulting from the retardation kinetics associated with the OER. The development of low-cost, high-activity, and long-lasting OER catalysts has emerged as a pivotal research area. Layered double hydroxides (LDHs) have garnered significant attention due to their suitability for use with base metals, which are cost-effective and exhibit enhanced activity. However, the current performance of LDHs OER catalysts is still far from meeting the demands of industrial applications, particularly in terms of their long-term stability. In this review, we provide an overview of the causes for the deactivation of LDHs OER catalysts and present an analysis of the various mechanisms employed to improve the stability of these catalysts, including the synthesis of LDH ultrathin nanosheets, adjustment of components and doping, dissolution and redeposition, defect creation and corrosion, and utilization of advanced carbon materials.

## 1. Introduction

The excessive exploitation and widespread utilization of traditional fossil fuels, including coal, petroleum, and natural gas, has resulted in a significant energy crisis and environmental contamination [1]. The search for new green and renewable energy sources is therefore crucial for achieving sustainable development goals. Hydrogen is considered to be one of the cleanest and most promising energy sources. Compared to the combustion of conventional fossil fuels, the burning of hydrogen only produces water, resulting in zero carbon emissions [2]. The most promising technology for producing green hydrogen is electrochemical water splitting with green electricity. Not only can it use green electricity from intermittent renewable energy sources such as solar and wind, it is also efficient, sustainable, and clean [3]. Electrochemical water decomposition consists of a hydrogen evolution reaction (HER) involving the transfer of two electrons and an oxygen evolution reaction (OER) with the transfer of four electrons. Importantly, the OER can also be used in other important electrochemical technologies, such as rechargeable metal–air fuel cells [4], electrocatalytic CO_2_/CO reductions [5,6], electrocatalytic nitrogen reductions [7], etc. Owing to the sluggish kinetics of four-electron OER and the high energy barriers for OER [8], excellent OER catalysts are being developed and optimized all the time. Noble metal-based compounds such as RuO_2_ [9] and IrO_2_ [10] exhibit remarkably efficient catalytic performance in alkaline solutions and even in acidic solutions. This is because hydrated hydroxyl complexes with a 6+ oxidation state are sufficiently stable and the coordination of the hydroxide is reversible. The high probability of protonation of the hydroxyl ligand allows for the removal of the hydroxide from the coordination sphere upon reduction back to the 4+ oxidation state [11,12]. Nevertheless, the high price and limited availability of resources have precluded the industrialization of noble metal-based catalysts. Consequently, the pursuit of cost-effective, highly efficient, and durable OER catalysts is imperative [13,14].

**Figure 1:**
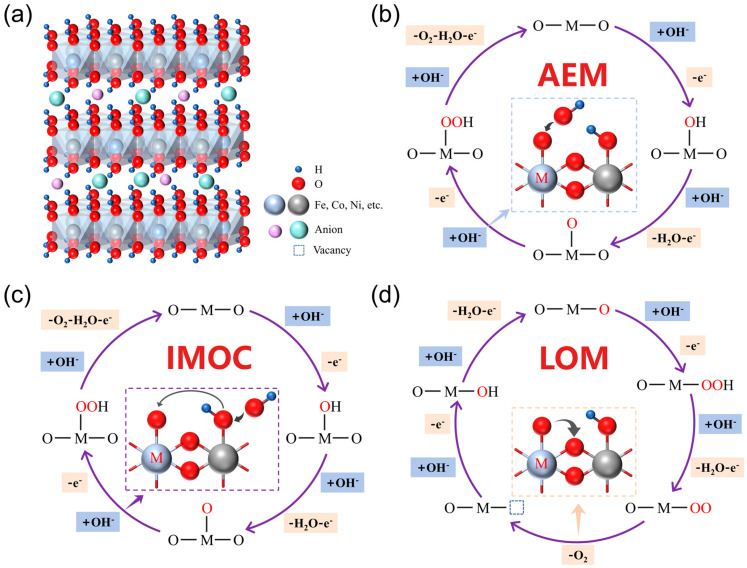
(**a**) Schematic structure of LDHs; (**b**) AEM reaction mechanism; (**c**) IMOC reaction mechanism; (**d**) LOM reaction mechanism.

Layered double hydroxides (LDHs) have attracted considerable attention due to their use of low-cost transition metals, tunable chemical composition and promising catalytic activity for OER. LDH is a hydrotalcite structure, schematically shown in Figure 1a. It has the generic formula ${\left[M_{1-x}^{2+}M_{x}^{3+}{\left(\mathrm{OH}\right)}_{2}\right]}^{x+}{\left[A_{x/p}^{p-}\right]}^{x-}\cdot yH_{2}O$, where M^2+^ and M^3+^ are metal cations of different valence states on the laminate (M^2+^: Mg^2+^, Fe^2+^, Co^2+^, Ni^2+^, Co^2+^, etc.; M^3+^: Al^3+^, Co^3+^, Fe^3+^, Mn^3+^, etc.). Metallic cations are bridged by the OH^−^ group, and A^p−^ indicates interlayer anions (Cl^−^, F^−^, etc.) neutralizing the excess positive charge of laminate [15]. The ratio of divalent to trivalent metal atoms determines the surface electronic states of LDHs, which in turn affect their catalytic activity. Up to now, three diverse reaction mechanisms have been mainly proposed for the OER on LDHs, i.e., the adsorbate evolution mechanism (AEM) [16,17], intramolecular oxygen coupling (IMOC) [18,19], and lattice oxygen-mediated mechanism (LOM) [20,21]. As shown in Figure 1b–d, the three mechanisms involve four OH^−^ and four electrons. The essence of the OER reaction in an alkaline solution is that OH^−^ ions are oxidized into O_2_, accompanied by the formation of many intermediates, such as M-OH, M-OOH, and M-O. All of these intermediates are actually associated with active sites on the catalysts, which can form chemical bonds more freely with the intermediates. The overall reaction path of AEM and IMOC is analogous, but the difference is that the active O in AEM produces strong adsorption of OH^−^ in the adsorbent to generate M-OOH, while the IMOC is adjacent to adsorption of the active O in the molecule and is directly coupled to transfer O-O free radicals. The activation of lattice oxygen in LOM enables the direct coupling of O-O radicals, which avoids the limitations imposed by the adsorption energy scaling relationship between M-OH and M-OOH via bypassing the generation of M-OOH [22]. Consequently, LOM can be understood as a way to address the mechanistic limitations of AEM. Wang et al. [23] elucidated the interactions between the applied potentials and competing kinetics of low-density Ni(M)OOH in a coupled reaction network consisting of three mechanisms. They further indicated that LOM on nickel-based LDHs can promote the activity of OER while depleting the lattice oxygen and causing the dissolution of the catalysts. The dissolution of the catalysts damages the stability of catalytic activity. Iron doping has been demonstrated to effectively stimulate AEM and attenuate LOM, improving activity and stability. The activity instability of the dominant LOM prevents investigators from pursuing highly active and durable OER catalysts [24].

The performance evaluation of OER catalysts in industry is based on two essential criteria: catalytic activity and stability. There is considerable debate as to whether there is a direct correlation between these two factors [25,26]. Over the past few years, the enhancement of OER activity for LDHs seems to have reached a plateau. However, studies on the enhancement of their stability are lacking and there is an overwhelming need for further development in this direction [27]. Stability requires a long-term evaluation of the electrochemical behavior of the catalyst, which on the one hand reveals the intrinsic changes in the catalyst during operation and on the other hand allows the development of optimization strategies. The importance of appropriate long-term stability testing is apparent [27]. Three methods are commonly employed in the laboratory to assess stability: cyclic voltammetry (CV), chronopotentiometry (CP), and chronoamperometry (CA). But the CV test method is less frequently employed in actual stability testing. This is because the industrial water electrolysis mainly uses a constant voltage mode or constant current mode, corresponding to CP and CA. And the water electrolysis is primarily conducted under a constant current, enabling precise control over the rate of hydrogen production. However, it is anticipated that there may be occasional discrepancies in the stability information derived from these approaches. Kuai et al. [28] observed that mixed nickel iron hydroxide (MNF) exhibited markedly disparate properties in CV and CA stability tests. In the CA test, MNF exhibited rapid deactivation, losing 24.1% of its current density after 15 h of reaction. In contrast, MNF demonstrated stable activity of over 500 cycles in the CV test. Consequently, it would be essential to employ a combination of these three methods in order to systematically acquire more dependable data regarding the durability of OER catalysts [29]. In order to estimate the long-term stability of OER catalysts, it is necessary to subject them to more rigorous testing conditions which are used in industrial water electrolysis, including higher current densities [30] and higher electrolyte concentrations [31].

**Figure 2:**
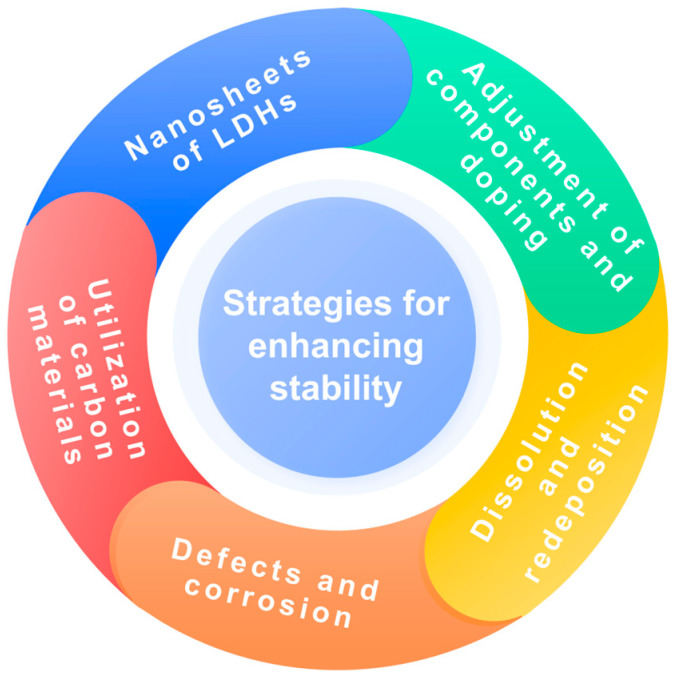
Schematic diagram of stability enhancement strategies for LDH OER catalysts.

Despite the multifaceted advancements in the activity and stability of LDH OER catalysts, there have been few reviews on the stability of LDH OER catalysts and their enhancement mechanisms. In this review, we examine the reasons behind the deactivation of LDH OER catalysts and the strategies employed to enhance their stability. The strategies mainly include nanosheets of LDHs, adjustment of components and doping, dissolution and redeposition, defects and corrosion, and utilization of advanced carbon materials (Figure 2). Moreover, this review offers insights for future studies on the stability of LDH OER catalysts and facilitates the rational design of long-lasting LDH OER catalysts.

## 2. Mechanisms for Deactivation of LDH OER Catalysts

**Figure 3:**
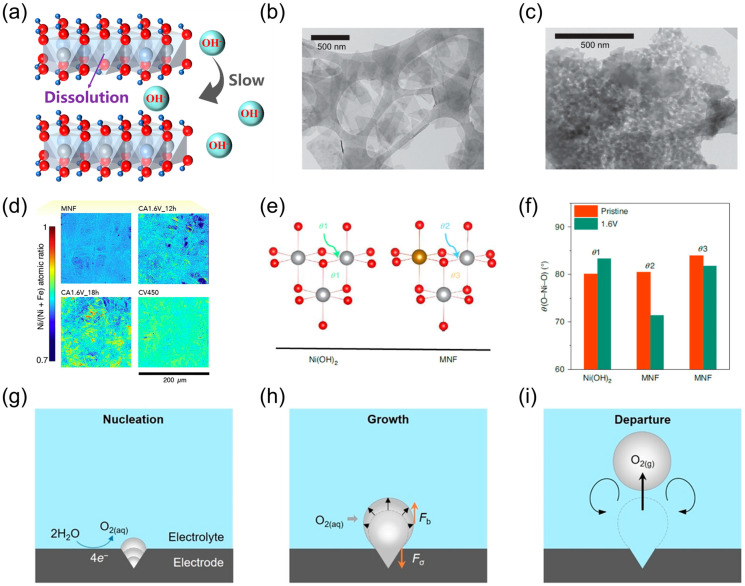
(**a**) Dissolution of LDH catalysts during alkaline water electrolysis is due to the inhibition of OH^−^ diffusion into interlayer space; (**b**,**c**) STEM imaging of representative regions of NiFe LDH samples before OER cycling (**b**) and after OER cycling (**c**); (**d**) XFM images depicting the mapping of atomic ratio Ni/(Ni + Fe) in the pristine MNF; (**e**) local structural changes in Ni(OH)_2_ and MNF at different angles; (**f**) local structural angles of Ni(OH)_2_ and MNF in pristine and OER conditions; (**g**) O_2_ bubble nucleation stage; (**h**) O_2_ bubble growth stage; (**i**) O_2_ bubble separation stage. Panel (**b**,**c**) reprinted with permission from Ref. [38], copyright 2023, Royal Society of Chemistry; panel (**d** **f**) reprinted with permission from Ref. [28], copyright 2020, The Author(s), under exclusive license to Springer Nature Limited; panel (**g** **i**) reprinted with permission from Ref. [39], copyright 2022, Wiley-VCH GmbH.

Investigating deactivation mechanisms is of great importance in explaining the instability of OER catalysts as well as in rationally designing highly durable catalysts [29]. For LDH OER catalysts, the most significant factor contributing to deactivation is the dissolution of active sites on surface [32,33]. The stability of LDHs is compromised in acidic or neutral electrolytes due to the proton (H^+^) acceptor properties of the hydroxides of transition metals, such as Fe, Co, Ni, etc., which are contained in LDHs. In acidic or neutral electrolytes, the concentration of hydroxide ions (OH^−^) is low and the transition metal hydroxides are the strongest bases in the system, making them susceptible to reacting with H^+^ [34,35]. Chen et al. [36] observed that NiFe LDH electrocatalyst experienced more pronounced dissolution of active sites in a neutral phosphate-buffered saline electrolyte than in an alkaline KOH electrolyte. The dissolution of LDHs during OER under alkaline conditions can be explained by the OER’s four electrons transfer process, which consumes four OH^−^ ions in the vicinity of the active site. When OH^−^ diffusion is not fast enough, a localized acidic microenvironment may be generated around the active sites, which leads to the solubilization of the active sites (Figure 3a) [37]. Chen et al. [36] conducted an investigation into the stability of NiFe LDH in OER. The findings indicated that the presence of a layered configuration in NiFe LDH hindered the rapid penetration of OH^−^ into the interlayer gaps. This resulted in the NiFe hydroxide acting as a proton acceptor and reacting with the H^+^ generated at the interlayer sites, which led to the dissolution of active sites and deactivation of LDHs for OER.

Another cause of instability in LDHs toward OER is phase transformation and segregation. This phase transformation during OER results in a reduction of the intrinsic activity of active sites. Daire Tyndall et al. [38] demonstrated the inherent instability of NiFe LDH-based OER catalysts. Their analysis revealed a gradual transition from the Ni(OH)_2_ phase to the NiOOH phase during the active life of the NiFe LDH-based OER catalysts. During OER, the Ni(OH)_2_ species would be oxidized into β-NiOOH or γ-NiOOH species, which act as the active sites for OER. Different from β-NiOOH, γ-NiOOH presents large interlayer distances, rich in water and alkali cations between layers, and a higher oxidation state of nickel. Therefore, the active Ni^3+^ species appearing during OER in the form of γ-NiOOH could reduce the OER overpotential [37]. The partial incorporation of Fe^3+^ in the Ni(OH)_2_ structure facilitates the oxidation of Ni(OH)_2_ species into γ-NiOOH species, leading to higher levels of activity for OER [38]. Therefore, the leaching of Fe atoms at relevant OER potentials is not conducive to the stabilization of γ-NiOOH species, resulting in the instability of the catalyst. The NiFe LDH sample before OER exhibited a lamellar morphology (Figure 3b). Its morphology underwent a significant change after 12 h of CP experiment for OER (Figure 3c). The stability of mixed Ni–Fe hydroxide (MNF) for OER under alkaline conditions was evaluated using CA and in situ XPS by Kuai et al. [28]. It was observed that the phase segregation became significant with increasing CA measurement time (Figure 3d). The distinct bond angles between pristine Ni(OH)_2_ and NiFe oxides (Figure 3e,f) indicate that Fe doping results in angular differences between the NiO_6_ and FeO_6_ octahedra, which in turn causes lattice distortions in the MNF. Furthermore, the redeposition of Fe also leads to deactivated phase segregation due to the formation of FeOOH-similar structures.

The attachment of OER catalysts to the substrate electrodes is achieved through various techniques, such as deposition, coating, and addition of adhesives. When the interaction between the catalysts and the substrate electrodes was diminished, the catalysts would become detached from the electrodes, potentially leading to instability within the OER system [40,41]. Due to the high porosity and high surface area of powder catalysts, the rapid evolution of oxygen on the surface may result in the detachment of weakly adherent powders from the electrodes [30]. Nevertheless, the incorporation of additives can improve the bonding between the catalyst powder and the substrate electrode. Andronescu et al. [42] conducted a comparative study of the OER activity and stability of NiFe LDH and a NiFe LDH–carbon matrix (NiFe/C) in alkaline electrolytes by using a CV test. The incorporation of the carbon matrix facilitated the immobilization of NiFe LDH on the substrate electrode, preventing the catalyst from falling off the substrate electrode surface.

Oxygen bubbles form in OER due to the limited solubility of molecular oxygen in the electrolyte. Bubbles are generally released at pores or cracks of the catalyst, beginning with nucleation, followed by growth and segregation (Figure 3g–i). Such behavior results in partial coverage of active electrocatalytic region and blockage of diffusion pathways for OH^−^ ions. Moreover, it negatively impacts the mechanical OH^−^ diffusion pathways of the catalyst on the electrode [39,43]. Therefore, it is imperative to ensure the timely completion of the bubble detachment process. Lu et al. [44] examined the OER stability of NiFe LDH by electrodepositing it on a glassy carbon (GC) electrode and performing CV measurements under alkaline conditions. It was observed that the catalyst was deactivated by bubble plugging during the CV scan, resulting in a decrease in the OER current density. However, the current density returned to the initial state after the removal of the bubbles by thorough water rinsing and subsequent nitrogen purging.

## 3. Stability Enhancement Strategies for LDH OER Catalysts

The stability of LDH OER catalysts is influenced by numerous factors, including the electronic structure [45], catalyst morphology, interlayer anions [46], dopant, type of substrate electrode, electrolyte pH [31,36], current density [47], reaction time [36], and temperature [31]. Modulation of these factors may facilitate alterations in diverse mechanisms, thereby stabilizing LDHs as OER catalysts. This section will provide a concise overview of the strategies employed to enhance the stability of LDH OER catalysts, which will serve as a foundation for the development of more durable LDH OER catalysts. The stability of some typical OER catalysts for LDHs is shown in Table 1.

### 3.1. Nanosheets of LDHs

**Figure 4:**
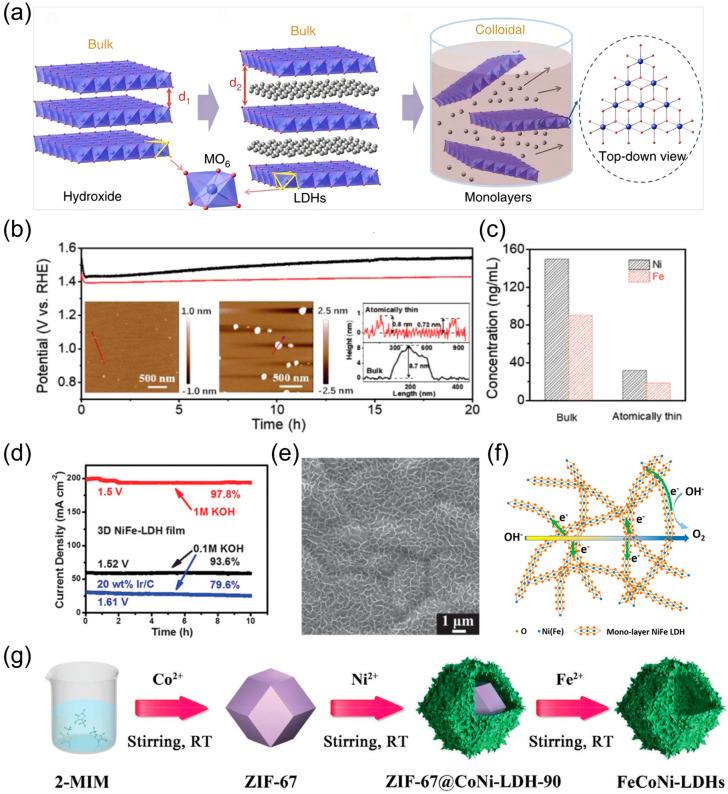
(**a**) Exfoliation process of LDH nanosheets; (**b**) stability of monolayer and multilayer NiFe LDHs for OER along with atomic force microscopy (AFM) images; (**c**) ICP-MS detection of dissolved nickel and iron ions; (**d**) stability test image of nickel–iron alloy LDH NP film and 20 wt% Ir/C; (**e**) SEM image of NiFe LDHs after 10 h stability test; (**f**) 3D microsphere formation diagram; (**g**) diagram of the formation process of FeCoNi-LDHs. Panel (**a**) reprinted with permission from Ref. [67], Copyright 2014, Springer Nature Limited; panel (**b**,**c**) reprinted with permission from Ref. [36], copyright 2019, WLEY-VCH Verlag GmbH & Co. KGaA, Weinheim, Germany; panel (**d**,**e**) reprinted with permission from Ref. [54], copyright 2024, Copyright Clearance Center, Inc. All rights reserved; panel (**f**) reprinted with permission from Ref. [70], copyright 2016, Elsevier B.V. All rights reserved; panel (**g**) reprinted with permission from Ref. [60], copyright 2021, Wiley-VCH GmbH.

Extracting multilayer LDH OER catalysts into atomically thin nanosheets reveals numerous active sites, which can effectively regulate the surface electronic structure. Additionally, the increased diffusion rate contributes to a reduction in bubble size and adhesion, thereby enhancing stability. Song et al. [67] employed a liquid-phase stripping technique to extract layered Ni-Fe, Ni-Co, and Co-Co LDHs into monolayer nanosheets (Figure 4a). Their findings revealed that the monolayer LDHs nanosheets exhibited reduced overpotentials and enhanced oxygen evolution activities, along with elevated stability (10 h) at 10 mA cm^−2^ compared to commercial IrO_2_. Chen et al. [36] utilized an ultrasonic stripping method to decompose the bulk multilayer NiFe LDH into atomically thin nanosheets under simulated operating conditions of alkaline electrolyzer in an industrial setting at a high temperature and high current density. The morphology of the monolayer of NiFe LDH remained unaltered after 20 h of electrolysis (Figure 4b,c), indicating increased catalytic stability. Fan et al. [68] successfully formed a Ni_0.75_V_0.25_ LDH monolayer through a standard hydrothermal incorporation process, whereby the abundant element V was introduced into Ni(OH)_2_. This resulted in a highly active catalyst with high electron-transferring capacity and abundant active sites. Furthermore, the stability of this material was found to be comparable to that of Ni_0.75_Fe_0.25_ (25 h). Lu et al. [54] prepared 3D films of vertically aligned NiFe nanoplates on 3D Ni foams (NiFe LDH NP) with a serrated skeleton and high porosity by hydrothermal treatment for 12 h at 120 °C. The films exhibited a low onset overpotential (~230 mV), a small Tafel slope (~50 mV dec^−1^), a high anodic current density (30 mA cm^−2^ of 280 mV), and outstanding electrochemical durability. The negligible degradation observed after 10 h of testing at constant overpotentials at 1.0 M KOH, along with the retention of 97.8% of the current density (Figure 4d), and the maintenance of its nanoplatelet array morphology (Figure 4e) demonstrates the robust stability of the material. Yu et al. [69] synthesized hierarchical hollow nanoprisms of ultrathin NiFe LDH via a self-templating approach. Upon hydrolysis of ferrous sulfate to consume the Ni precursor, a monolayer of NiFe LDH nanosheets was generated on its surface and the internal template was converted to an active substance while dissolving. The final synthesized NiFe LDH hollow nanoprisms exhibit a large specific surface area, high OER electrocatalytic activity, and excellent stability. After 1000 cycles of the voltammetry test, the change in the overpotential at 10 mA cm^−2^ was minimal. Li et al. [70] successfully obtained NiFe LDH 3D microspheres (Figure 4f) with atomically thin structures through a hydrothermal treatment process utilizing a solvent mixture of deionized water and butanol. The synergistic effect of the released carbonate anion and butanol was identified as the key factor controlling the generation of atomically thin structures. In contrast, only deionized water was used as the solvent for the preparation of 2D NiFe LDH. ${\mathrm{CO}}_{3}^{2-}$ can interact with the positively charged LDH layer due via electrostatic interactions, whereas butanol can selectively absorb onto the {001} facets of LDHs to reduce surface energy. It is evident that the simultaneous presence of ${\mathrm{CO}}_{3}^{2-}$ and butanol can expand the interlayer spacing of LDHs, thereby facilitating the formation of nanosheets with atomic thickness. A comparison of NiFe LDH 3D microspheres with an atomically thin layer structure with 2D NiFe LDH reveals greater stability of the former. Zhang et al. [60] developed a facile room-temperature stirring strategy using a metal framework as a precursor to synthesize FeCoNi-LDHs nanocages comprising ultrathin nanosheets (Figure 4g). These nanocages exhibited markedly extended stability toward OER. The optimized FeCoNi-LDHs nanocages demonstrated remarkable stability, retaining 96.4% of the initial current density after 100 h of OER operation at a constant voltage. In comparison, the CoNi-LDH nanocages and the benchmark IrO_2_ exhibited a notable decline in performance. The incorporation of Fe in FeCoNi-LDHs nanocages was evidenced to increase the interlayer spacing and provide additional active sites for OER. Additionally, the nanomorphology, wide surface area, and porosity of FeCoNi-LDHs nanocages promoted long-term stability. Yu et al. [71] employed a two-step synthesis to grow few-layer NiFe LDH nanosheets on Cu nanowire cores, thereby fabricating highly efficient 3D bulk catalysts with core–shell nanostructures. An overpotential of only 199 mV is sufficient to deliver an OER of 10 mA cm^−2^ at 1.0 M KOH. The device exhibits stable overpotentials for 48 h at 100 mA cm^−2^. Moreover, its morphology remains largely unaltered even after the OER, which is indicative of its excellent stability.

Nanoprocessing of LDH OER catalysts also encompasses nanowire arrays [72], nanotube arrays [73], nanocone arrays [74], heterostructures [56], hollow nanoparticles [75], hollow sphere [76] and other forms. Nanostructures can buffer volume changes during redox cycling and virtually prevent structural deformation while promoting oxygen evolution [3]. Morphological alterations are made to expose additional active sites and reinforce the interlayer structure, thereby enhancing catalytic activity and stability to a certain extent.

### 3.2. Adjustment of Components and Doping

The stability of LDH OER catalysts can be influenced by the ratios of metal content. Yang et al. [77] prepared ordered nanosheet structures of NiFe LDH with Fe contents ranging from 0 to 25% by complexation. It was determined that samples containing 15% Fe exhibited the highest anodic current density, with the anodic potential of the 15% Fe-doped samples demonstrating consistent stabilization at 1.49 V for 15 h in the CP test, which surpassed that of the commercially available 20% Ir/C. Zeng et al. [78] investigated the impact of the composition of NiFe bimetallic substrates on OER catalytic activity and stability. It was observed that the OER performance of hydrothermally treated NiFe LDH, synthesized using a NiFe substrate containing 60% Fe in a 1.0 M KOH solution, exhibited superior results compared to those obtained from NiFe LDH prepared with substrates containing 5%, 25%, and 95% Fe content. The former demonstrated an overpotential of 270 mV at 10 mA cm^−2^ for 14 h without a notable decline. This outcome substantiated the catalytic durability of the NiFe substrate with a Fe content of 60% and validated the hypothesis that the intrinsic catalytic activity can be enhanced by the optimization of the metal atom ratio in the LDHs.

**Figure 5:**
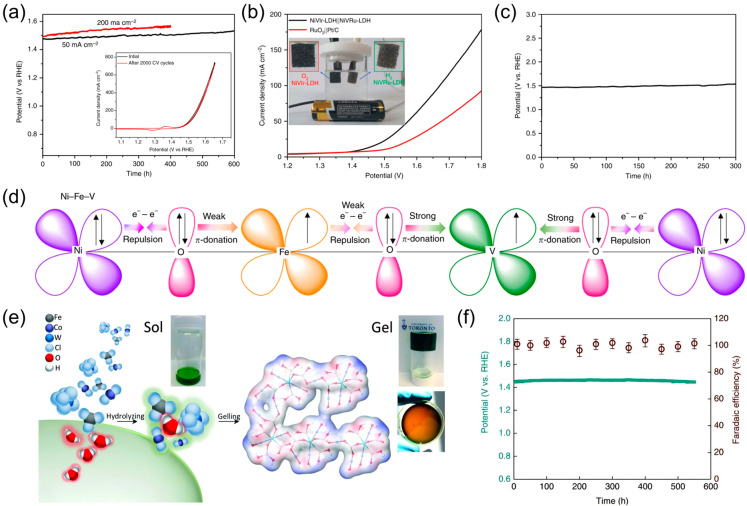
(**a**) CP test images of NiVIr-LDH at different current densities. Inset: NiVIr-LDH polarization curves after 2000 CV cycling tests (the peak between 1.3 V and 1.4 V is due to the oxidation of Ni^2+^ to Ni^3+^ in the catalyst [83]); (**b**) NiVIr-LDH||NiVRu-LDH and RuO_2_||Pt/C polarization curves for overall water electrolysis; (**c**) NiVIr-LDH||NiVRu-LDH CP test for overall water electrolysis at 10 mA cm^−2^; (**d**) schematic of electron coupling in Ni_3_Fe_0.5_V_0.5_; (**e**) schematic diagram of the preparation process of the gelatinized structure of G-FeCoW. (**f**) CP tests of G-FeCoW oxyhydroxides at 30 mA cm^−2^ and corresponding Faraday efficiencies. Panel (**a** **c**) reprinted with permission from Ref. [61], copyright 2019, The Author(s), under exclusive license to Springer Nature Limited; panel (**d**) reprinted with permission from Ref. [80], copyright 2018, The Author(s), under exclusive license to Springer Nature Limited; panel (**e**,**f**) reprinted with permission from Ref. [62], copyright 2016, The American Association for the Advancement of Science.

Doping modification has the potential to significantly modify the bonding energy of the intermediates during OER, thereby improving the performance of OER catalysts [79]. Loading noble metal nanostructures on LDH OER catalysts represents a valid method to enhance the electrocatalytic performance. This approach not only utilizes a minimal amount of noble metal but also improves the stability of the catalysts. Wang et al. [61] synthesized NiVRu LDH and NiVIr LDH catalysts on nickel foam by doping Ru or Ir into NiV LDH via a one-pot hydrothermal method. The doping of Ru or Ir induced changes in the local atomic structure of Ni and V as well as the presence of V vacancies. Furthermore, the activation energy of the OER rate-determining step was optimized by Ru or Ir doping. NiVIr LDH necessitated merely 180 mV overvoltage to attain 10 mA cm^−2^ and presented a stable OER performance for 400 h at 200 mA cm^−2^ (Figure 5a). In an alkaline electrolyzer, the utilization of NiVRu LDH and NiVIr LDH as the cathode and anode, respectively, resulted in a cell voltage requirement of only 1.42 V to achieve a current density of 10 mA cm^−2^, while maintaining high activity for a duration of 300 h (Figure 5b,c). Jiang et al. [80] co-doped Fe and V into Ni(OH)_2_ to obtain Ni_3_Fe_0.5_V_0.5_ catalysts, in which Fe and V modulate the local coordination environments and electronic structures of the catalysts (Figure 5d). In addition, Ni_3_Fe_0.5_V_0.5_/CFP electrodes demonstrated robust stability over a period of 60 h during electrolysis under 100 mA cm^−2^. Peng et al. [81] doped W, Mo, Nb, Ta, and Re elements into NiFe and FeCo to form NiFeX and FeCoX metal hydroxides. They utilized the principle that metals with higher charges can adjust the transition energy of metals with lower charges, thereby achieving enhanced catalytic OER performance. In water electrolysis using a NiFeMo catalyst as the anode and a commercial Ru electrode as the cathode, the cell voltage was stable for 12 h at 80~85 °C and 2 MPa, with a high current density of 300 mA cm^−2^ retained. Zhang et al. [62] implemented a room-temperature sol–gel methodology to generate gelation hydroxides (G-FeCoW) with a uniform distribution of three metal elements (Figure 5e). The synergistic interactions among W, Fe, and Co resulted in the formation of favorable local coordination environments and electronic structures, which strengthened the OER performance. Doping Fe and W onto the surface of CoOOH is conducive to improving the energetics of OER intermediates, thus regulating the catalytic activity of the materials. Moreover, the incorporation of a W atom at a Co^4+^ site results in the displacement of protons from W towards oxygen at Co sites, and compressive strain on the surrounding Co sites due to the larger size of W atoms. These geometric and electronic changes induce a favorable direct O_2_ mechanism for OER with a low theoretical overpotential. The G-FeCoW catalyst deposited on gold-plated Ni foams was subjected to water oxidation experiments for a period of 550 h at 30 mA cm^−2^ (Figure 5f). During this time, no considerable increase in potential was observed, indicative of exceptional stability. In addition, Feng et al. [82] incorporated cobalt into NiFe LDH to facilitate iron redeposition and utilized electrodeposition to embed borate into the NiCoFe LDH layer. The catalyst exhibited excellent stability, retaining its activity for 1000 h during the OER test at 10 mA cm^−2^ and pH 14, without any observable degradation.

**Figure 6:**
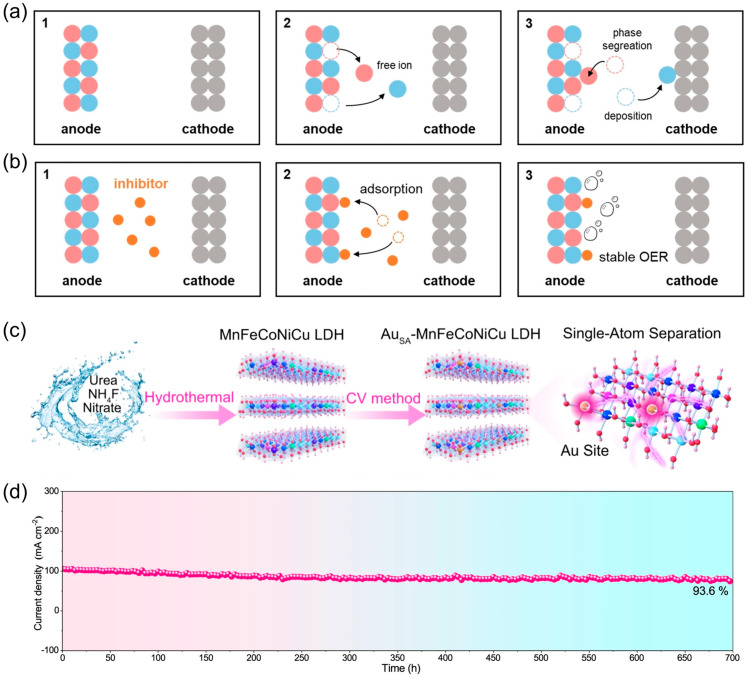
Schematic diagram of active site dissolution (**a**) and inhibitor mechanism (**b**) of NiFe-LDH in the electrolytes containing different inhibitor; (**c**) synthesis schematic of Au_SA_-MnFeCoNiCu LDH; (**d**) stability test of Au_SA_-MnFeCoNiCu LDH. Panel (**a**,**b**) reprinted with permission from Ref. [86], copyright 2024, Wiley-VCH GmbH; panel (**c**,**d**) reprinted with permission from Ref. [64], copyright 2023, The Author(s), under exclusive license to Springer Nature Limited.

The intercalation of diverse anions between the layers of LDHs OER catalysts modifies the electronic structure of the surface metal sites and influences layer spacing. Accordingly, the anions intercalated in the LDHs could affect the stability of OER catalysts. Dong et al. [84] synthesized NiFe LDHs with varying layer spacings by incorporating dibasic carboxylic acids with disparate alkyl chain lengths into LDHs via a one-pot co-precipitation approach. Comparisons revealed that the catalytic performance of the resulting NiFe LDHs modified with an octanoate anion is superior to the commercial RuO_2_ catalyst in a borate electrolyte (0.1 M, pH = 9.2). Furthermore, the former catalyst exhibited excellent OER stability, as evidenced by its sustained activity over a 24 h period. Zhang et al. [85] employed a process of embedding benzoate anion (BZ) into NiFe LDH nanosheet arrays to create a sheet catalyst on carbon cloth (BZ-NiFe-LDH/CC). The BZ anion has demonstrated resistance to the detrimental electrochemical effects of chloride ions, as observed during the electrolysis of seawater. Moreover, this anion has been shown to enhance the interlayer spacing of NiFe-LDH, thus facilitating electrolyte penetration and diffusion. The BZ-NiFe-LDH/CC electrode exhibits excellent electrochemical stability, as evidenced by 100 h of electrolysis at a high current density of 500 mA cm^−2^. Liu et al. [86] discovered that a number of oxygen anions, including phosphate, sulfate, borate, and carbonate, can be employed to safeguard NiFe-LDHs from degradation (Figure 6a,b). For instance, phosphate can be adsorbed on NiFe-LDH OER electrodes during electrolysis, acting as an inhibitor that reduces the dissolution of Ni atoms. This results in a 25-fold reduction in the performance decay rate. In order to establish a baseline for the performance of an industrial alkaline water electrolyzer, a two-electrode system was constructed with nickel foam serving as an OER catalyst for electrolysis at 400 mA cm^−2^ at 80 °C. The system proved capable of stable operation for 100 h.

An additional method for modifying the composition is to incorporate a greater number of transition metal elements, thereby creating high-entropy catalysts. The enhanced stability of high-entropy materials can be attributed to entropic stabilization effects [87,88] and slow diffusion effects [89,90], which collectively prevent phase transitions and metal leaching [91]. Yao et al. [63] reported the synthesis of FeCoNiCuZn-LDHs (HE-LDHs/CC) on carbon cloth via a hydrothermal process. Subsequently, some of the metal cations were removed through chemical etching, creating cation vacancies (HE-LDHs-V^+^/CC). It has the ability to enhance the intrinsic activity and optimize the adsorption energy of intermediates involved in OER. Notably, the HE-LDHs-V^+^/CC samples displayed exceptional stability, retaining their functionality for 200 h with a current density decay of only 3.8% in the CA test. Chen et al. [92] carried out the synthesis of CoCrFeNiMo LDH arrays in situ on CoCrFeNiMo high-entropy alloys using a one-step etching method. The catalytic performance was augmented due to the porous morphology of the resulting materials with extensive surfaces, which ensured efficacious mass transfer and provided a substantial number of active metal sites. Moreover, the OER performance of catalysts exhibited minimal fluctuations over an extended period under 100 mA cm^−2^, suggesting its durability for OER. Wang et al. [64] prepared a high-entropy MnFeCoNiCu LDH decorated with Au mono-atoms and O vacancies (Au_SA_-MnFeCoNiCuLDH) by combining hydrothermal and electrochemical deposition methods (Figure 6c). The presence of O^2−^ as indicated by both ^18^O isotope-labeled mass spectrometry and in situ Raman spectroscopy highlights the transition of the OER mechanism from AEM to LOM. DFT calculations further confirmed that the Au mono-atoms and oxygen vacancies synergistically weaken the metal–oxygen bonds, facilitating the activation of lattice oxygen and thereby reducing the energy barrier for water oxidation via the LOM mechanism. Most notably, the current density exhibited minimal decay at 6.4%, following 700 h of CA testing at a potential of 1.53 V (vs. RHE). This illustrated a prominent long-term stability, surpassing that of the majority of LDH OER catalysts (Figure 6d).

### 3.3. Dissolution and Redeposition

**Figure 7:**
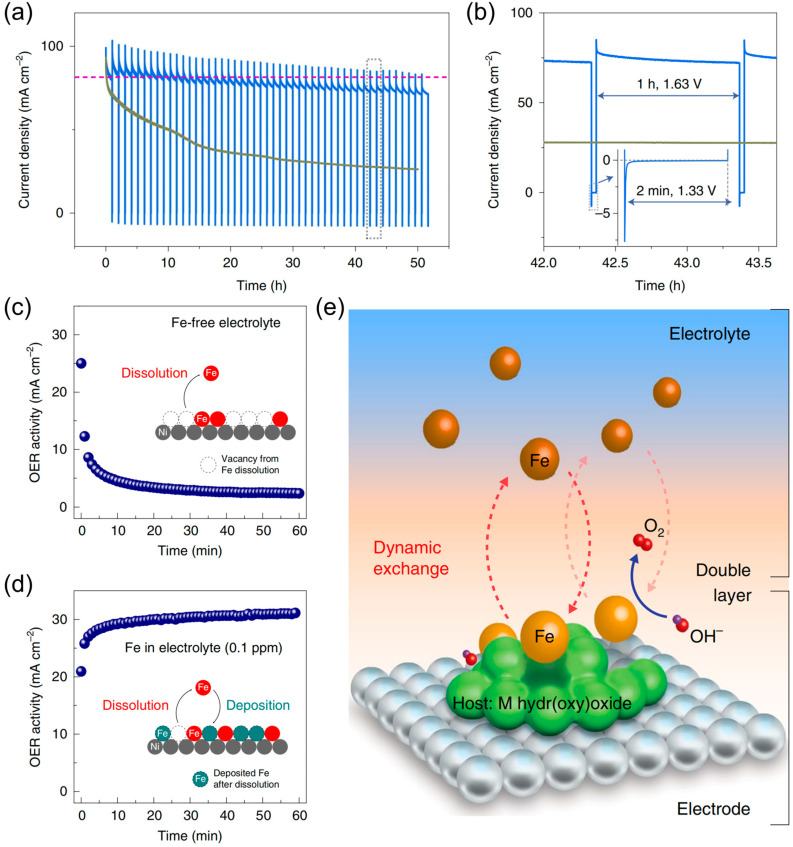
(**a**) Long-term stability testing of bulk MNF. Green line: continuous CA test. Blue line: intermittent CA test. (**b**) Localized enlargement of panel (**a**); (**c**) CP test at 1.7V in ‘Fe free’ electrolyte; (**d**) CP test at 1.7 V in an electrolyte containing 0.1 ppm Fe; (**e**) schematic representation of ‘dynamically stabilized’ active site/host pairs at the electrode/electrolyte interface. Panel (**a**,**b**) reprinted with permission from Ref. [28], copyright 2020, The Author(s), under exclusive license to Springer Nature Limited; panel (**c** **e**) reprinted with permission from Ref. [93], copyright 2020, The Author(s), under exclusive license to Springer Nature Limited.

As previously indicated, the dissolution of active metal sites can result in the destabilization of LDH OER catalysts, particularly when lattice oxygen is involved in the OER via the LOM mechanism. Dissolution–redeposition represents an effective strategy for addressing catalyst instability issues. Revising the operational parameters for managing the dissolution–redeposition procedure has the potential to enhance the longevity of the catalyst. In a study conducted by Kuai et al. [28], it was observed that the local structure of mixed nickel–iron hydroxide underwent significant distortion when subjected to a long-term OER CA test. This distortion, particularly at high potentials, accelerated the dissolution and segregation of the metal atoms, resulting in the emergence of an interfacial segregation between FeOOH and NiOOH. Further investigation determined that the phase separation of NiFe hydroxides is reversible in the potential range between the potential for OER and the potential for catalytic reduction. Accordingly, an intermittent reduction method was devised to produce oxygen at 1.63 V for 1 h, followed by catalytic reduction at 1.33 V for an additional 2 min. This procedure was employed in CA tests to recover NiFe hydroxides and improve the durability of the catalysts (Figure 7a,b). The underlying principle of this process is that during the OER reaction, the dissolved irons are redeposited at the edge position of NiOOH, which results in phase segregation. Conversely, in the reduced state, the uniform redeposition of dissolved metal ions serves to mitigate the phase segregation. Chung et al. [93] advanced the concept of dynamic stability, elucidating its underlying principle as the dissolution–redeposition of metal ions. The researchers employed hydroxide clusters (MO_x_H_y_, M = Fe, Co, Ni) as hosts, and observed that Fe was selectively adsorbed onto the hydroxide matrix following the introduction of 0.1 ppm Fe ions into the alkaline electrolyte. They established dynamically stabilized Fe-active sites by balancing the rate of Fe solubilization and the rate of redeposition (Figure 7e). The incorporation of iron ions into the electrolysis process presented a significant enhancement in stability (Figure 7c,d). This observation underscores the critical role of regulating the dissolution and redeposition process for achieving long-term stability.

### 3.4. Defect Creation and Corrosion

**Figure 8:**
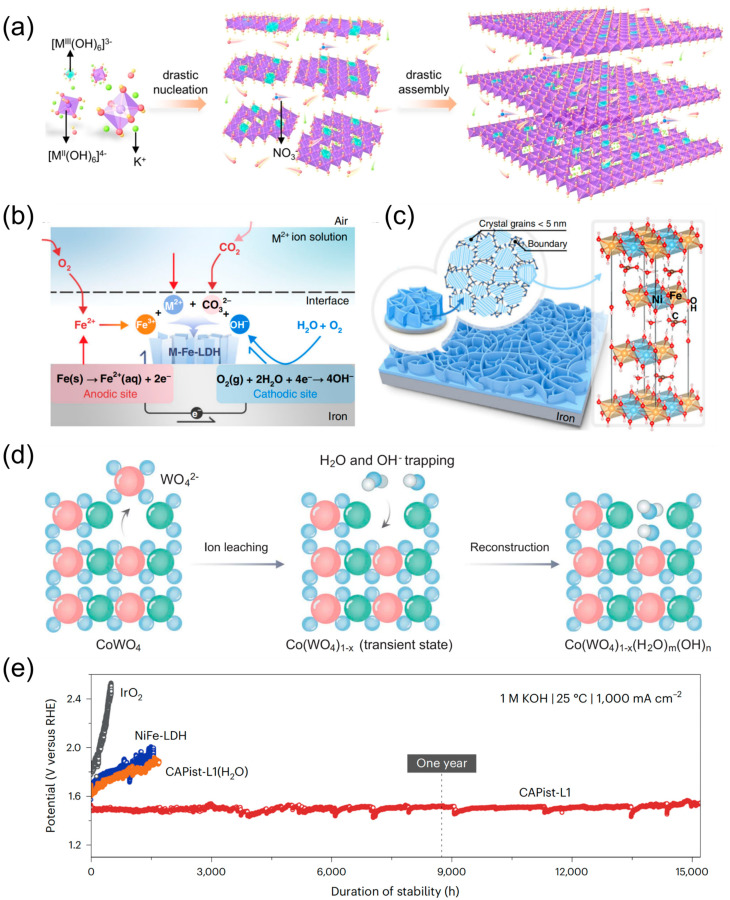
(**a**) Schematic depiction for precipitation of LDHs under drastic non-equilibrium conditions; (**b**) corrosion processes in iron substrates; (**c**) formation of LDH nanosheet arrays on iron substrates; (**d**) crystallographic process for the formation of CWO-del-48 by alkali treatment, the red, green, blue and white atoms represents W, Co, O and H atoms respectively; (**e**) long-term stability testing of CAPist-L1 at 1000 mA cm^−2^ for OER. Panel (**a**) reprinted with permission from Ref. [37], copyright 2023, Wiley-VCH GmbH; panel (**b**,**c**) reprinted with permission from Ref. [65], copyright 2018, The Author(s), under exclusive license to Springer Nature Limited; panel (**d**) reprinted with permission from Ref. [66], copyright 2024, The American Association for the Advancement of Science; panel (**e**) reprinted with permission from Ref. [95], copyright 2024, The Author{s), under exclusive license to Springer Nature Limited.

Defects significantly impact the stability of the LDHs catalysts, particularly with regard to vacancies, which contribute to the change in binding energy between metal atoms and oxygen atoms. This maximizes the optimization of the binding energy for OER intermediates, resulting in an improvement in the overall kinetics of OER [52]. The dissolution of LDH catalysts was also markedly suppressed under OER conditions, which thus enhanced the stability of electrocatalysis. Peng et al. [52] prepared thin layers of NiFe LDH doped with Zn^2+^, Al^3+^, Zn^2+^/Al^3+^ via a controlled synthesis route. Subsequently, cationic vacancies of M^2+^ and M^3+^ were produced by alkaline etching of Zn and Al atoms from the LDH using 1 M KOH solution. Observations indicate that the structure of LDH remains intact after selectively etching Zn and Al, without any collapse occurring around the removed metal atoms. Additionally, it was demonstrated that the constructed cationic vacancies may exist as single-atom vacancies. The formation of cationic vacancies impedes the dissolution of the metal atoms and the occurrence of undesirable phase segregation. Comparative stability demonstrated that NiFe LDH with Ni vacancies and Ni and Fe double vacancies exhibited degradation rates of only 2.19% and 2.22% after 1000 CV cycles. Zhang et al. [53] synthesized monolayers of NiFe LDH via co-precipitation. The accelerated formation and scaling of monolayer LDH nanosheets was then successfully achieved by increasing the concentration of alkali and metal ions by a factor of 10. Concurrently, the irregular porous structure was observed as a result of the formamide decomposition on the surface of the nanosheet. The presence of oxygen and cation vacancies leads to distortion of the LDH nanosheets and modification of their electronic properties. The CA tests revealed that at an overpotential of η = 230 mV, the OER activity could be sustained for 100 h when the electrolyte was replenished every 20 h. Li et al. [37] described a non-equilibrium precipitation methodology for the fabrication of LDH OER catalysts possessing a crystal structure characterized by significant disorder (d-NiFe-LDH) (Figure 8a). The catalyst generates a high density of cationic vacancies due to non-equilibrium nucleation. Simultaneously, in the 1 M KOH reaction environment, the rapid formation of LDH results in K^+^ doping, thereby creating a localized base-rich environment in proximity to the active site. The high density of bi-cationic and multi-cationic defects trap a greater number of base ions. The close proximity of these base ions to neighboring OH^−^ in the local environment promotes a rapid and efficient movement of OH^−^ from the electrolyte to and between active sites. This process enables the overcoming of the creation of localized acidic environments and the disintegration of active sites during the OER process. Significantly, the d-NiFe-LDH also demonstrates impressive durability, with a slight rise in potential from 1.41 V to 1.48 V over 900 h of continuous operation in the long-term CP test, in contrast to the rapid decline of activity observed for ordinary NiFe-LDH. In addition, Xie et al. [94] introduced a substantial number of uniform sub 3 nm nanopores on 1.5 nm thick NiFe LDH nanosheets through a technique that combines etching and aging, which markedly enhanced the OER activity and stability.

Corrosion exchange engineering has garnered the interest of numerous researchers. Liu et al. [65] immersed affordable iron substrates in an aqueous solution containing divalent cations (e.g., Ni^2+^, Co^2+^, Mn^2+^, Mg^2+^) at a concentration of 100 μmol L^−1^, which resulted in the creation of well-oriented, grain-boundary-enriched arrays of nanosheets on the iron substrates (Figure 8b,c). The binder-free nano-array structure is conducive to avoiding irregular aggregation of catalytically active phases, exposing catalytically active sites, and facilitating mass transfer during OER. The catalyst also showed enhanced stability, allowing electrocatalytic OER to be carried out for 5000 h in 1 M KOH solution at a large current density of 1000 mA cm^−2^ and 1050 h in a 10 M KOH high-concentration solution. As reported by Ranit et al. [66], the delamination of cobalt tungstates has been observed to stabilize the oxide and water-hydroxyl networks, thereby enhancing the acidic OER activity and durability. The researchers achieved delamination through the integration of high oxidation state sacrificial elements (e.g., tungsten) into the crystal structure of CoWO_4_ (CWO). Subsequently, the ${\mathrm{WO}}_{4}^{2-}$ ions were substituted with OH^−^ and H_2_O through a cation-exchange strategy, resulting in the formation of CWO-del-48 (Figure 8d). The formation of a thermodynamically unfavorable barrier for the solubilization of cobalt ions, comprised of OH^−^ and H_2_O trapped in the cobalt oxide corrosion network, significantly reduces the solubilization of cobalt ions in acids, in comparison to un-delaminated Co_3_O_4_. The delaminated CoWO_4_ catalyst displayed long-term stability, operating stably for 278 h at 0.2 A cm^−2^ and 608 h at 1.0 A cm^−2^ in a proton exchange membrane water electrolysis (PEMWE) system at a temperature of 80 °C. These results demonstrate the potential for the catalyst to be utilized in long-term applications at large current densities.

Li et al. [95] have successfully devised a single-step technique for seed-assisted heterogeneous nucleation (25 °C, 24 h) to fabricate an electrocatalyst based on nickel and iron (CAPist-L1), which possesses a distinctive structure comprising an intermediate layer. The intermediate layer, formed by the corrosion of insoluble nanoparticles, exhibited sufficient mechanical stability to resist the adhesive force of oxygen bubbles and catalyst shedding [96]. The OER activity of the CAPist-L1 catalyst was found to be significantly high, demonstrating low overpotentials of 220 ± 4.5 mV at a current density of 1000 mA cm^−2^ and 283 ± 12.7 mV at a higher current density of 5000 mA cm^−2^. Notably, the CAPist-L1 catalyst displayed remarkable durability, operating for 15,200 h in the CP test conducted at 1 M KOH, 25 °C, and 1000 mA cm^−2^ (Figure 8e). Its stability was particularly impressive, exceeding that of NiFe LDH and commercial IrO_2_.

### 3.5. Utilization of Advanced Carbon Materials

**Figure 9:**
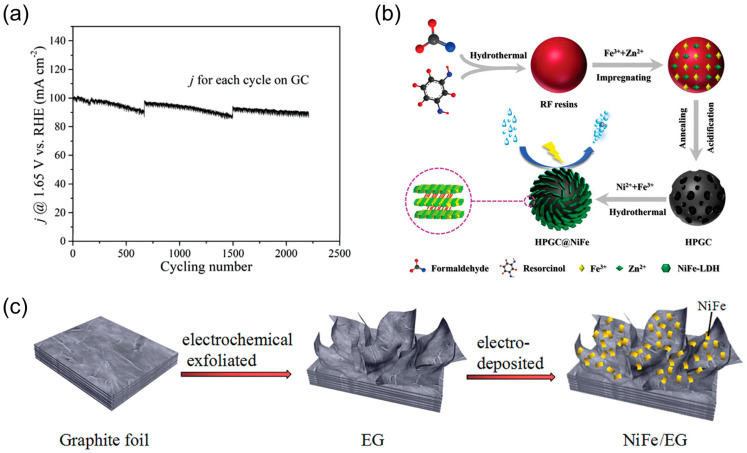
(**a**) CV cycling test for NiFe/C on GC; (**b**) schematic of HPGC@NiFe fabrication process; (**c**) manufacturing process of the NiFe/EG electrode. Panel (**a**) reprinted with permission from Ref. [98], copyright 2017, WILEY-VCH Verlag GmbH & Co. KGaA, Weinheim; panel (**b**) reprinted with permission from Ref. [99], copyright 2024, Copyright Clearance Center, Inc. All rights reserved; panel (**c**) reprinted with permission from Ref. [100], copyright 2024, Copyright Clearance Center, Inc. All rights reserved.

The presence of a hydrophobic interfacial layer between the prepared carbon material substrate and the LDH OER catalysts modulates the local electronic structure of the cation, thereby influencing the growth of the catalyst. It promotes effective dispersion and serves as a strong coupling interface between the collector electrode and the active phase, thereby enhancing the OER performance [3]. Liu et al. [97] proposed a hydrothermal method for the direct growth of NiFe-LDH on flexible single-walled carbon nanotube (SWNT) films, resulting in the formation of NiFe-LDH@SWNT. The in-situ growth process ensures that NiFe-LDH is efficiently deposited onto the surface of each SWNT bundle unit, forming a distinctive integration of LDH and SWNT while circumventing the deactivation of the active ingredient. The NiFe-LDH@SWNT material exhibits high durability, as evidenced by its ability to retain OER activity for 20 h in a CA test. Yin et al. [98] successfully developed an efficient NiFe-LDH/C OER catalyst through a one-pot synthesis approach, employing precursors that are a molecular mixture of metal and carbon sources. During the solvothermal synthesis of NiFe-LDH, the organic ligand is catalyzed by Fe to decompose and transform into amorphous carbon with graphite nanodomains, and the localized growth of NiFe-LDH and C in a single fragment result in a fully integrated amorphous NiFe-LDH/C nanohybrid. The doping of carbon nanodomains not only enhances the electronic characteristics of this composite but also promotes the increased accessibility of surface redox sites. In the CP test, the samples sustained a current density of 40 mA cm^−2^ with a loss of activity of less than 5% over a period of 28 h. In the CV test, following 2000 cycles, a slight decrease in current density was observed, from 100 mA cm^−2^ to 95 mA cm^−2^, indicative of enhanced stability (Figure 9a). Ni et al. [99] constructed a core–shell structure of hierarchically porous graphitized carbon (HPGC)-loaded NiFe LDH (Figure 9b), which exhibited greater stability. The synthesis commenced with the preparation of monodisperse phenolic (RF) resin spheres of 670 nm from resorcinol and formaldehyde. Subsequently, HPGC nanospheres were obtained through the activation/graphitization of RF resin using zinc salts and trivalent iron. Ultimately, NiFe LDHs were grown on HPGC employing a single-step hydrothermal technique. The advantage of HPGC is that it promotes charge transfer, while the porous structure facilitates mass transfer and gas diffusion. Concomitantly, the vertical growth of NiFe LDHs facilitates the exposure of active sites and the stacking of LDH sheets during electrochemical processes. Stability tests were conducted using CP and CA, respectively, with no discernible change in current density or 50 h at 1.495 V when the current density was maintained at 10 mA cm^−2^. Upon increasing the current density to 50 mA cm^−2^, the potential exhibited stability for a period of 24 h.

Combining nanostructured NiFe hydroxide with exfoliated graphite also results in the exposure of a greater number of active sites, which facilitates electron transfer and thus improves OER properties. In a seminal work, Yet et al. [100] initially produced partially exfoliated graphite through electrochemical processing, following which they electrodeposited amorphous NiFe hydroxide nanosheets onto a 3D exfoliated graphite foil substrate (EG) (Figure 9c). The NiFe/EG composites that have been prepared demonstrate excellent electrical conductivity as well as a significant catalyst deposition surface area and pathways for oxygen diffusion. The potential remained unaltered at 1.48 V for 100 h at 10 mA cm^−2^ in the NiFe/EG electrode CP test. At a current density of 500 mA cm^−2^, the potential exhibited a slight increase of 20 mV after 48 h, with no discernible change in the structure. This demonstrated that the catalyst exhibited exceptional stability and physico-mechanical properties. Additionally, Ruan et al. [101] prepared NiFe/GP electrodes via the electrodeposition of nickel–iron hydroxide on graphite paper (GP) using a constant potential pulse technique. The combination of graphite and NiFeOH offers a unique advantage: the graphite substrate enhances electron transfer, while NiFeOH provides more active sites. The catalytic electrodes were subjected to 1000 cycles of CV cycling and OER testing at 320 mV overpotential for 20,000 s. The electrode morphology remained unchanged, indicating its long-term durability.

## 4. Conclusions and Perspectives

OER presents a significant challenge to water electrolysis, due to the slowness of the associated chemical kinetics and the necessity for high overpotentials. Consequently, related catalysts were optimized and developed. Among them, LDHs are widely employed in OER due to their utilization of transition metal, which are cost-effective and exemplify notable activity and stability. In this review, we examine three distinct reaction mechanisms for LDH OER catalysts, with particular attention paid to the LOM mechanism, which accounts for catalyst dissolution during OER due to lattice oxygen depletion. Moreover, the underlying factors that lead to the deactivation of LDHs, including dissolution, phase transition and separation, falling off from substrates, and bubble clogging, are elucidated in a concise manner. A variety of stability-enhancing mechanisms for LDH OER catalysts are presented. These include nanosheets of LDHs, adjustment of components and doping, dissolution and redeposition, defect creation and corrosion, and utilization of advanced carbon materials. The introduction of these additional components serves to enhance the overall stability of the catalysts. It is possible that a combination of these mechanisms may yield unanticipated outcomes.

While high stability is a primary criterion for assessing the performance of catalysts, the evaluation system for catalyst stability remains a subject for improvement. Currently, there is no definitive criterion for differentiating between stable and unstable catalysts. Some researchers have developed LDH OER catalysts for stable operation under high current density and high electrolyte concentration with the aim of establishing benchmarks for industrialization. However, there is still a considerable distance to travel before specific industrial references can be made. Industrialized OER catalysts are employed in a variety of systems, including alkaline electrolyzers, PEM electrolyzers, and AEM electrolyzers. Therefore, the specific environment, substrate type, electrolyte impurities, and other factors can significantly impact the suitability and efficacy of OER catalysts for LDHs. The future research and development of OER catalysts for LDHs should be a collaborative effort between laboratory workers and corporate personnel. This collaboration should have the following objectives: to enhance catalyst activity and stability, increase achievable current densities and recoveries, and to strive for industrial applications to promote solutions to energy and environmental problems.

## Figures and Tables

**Table 1:** Stability of typical LDH OER catalysts.

Catalysts	η@10 mA cm^−2^/mV	Stability/h	References
FeNi-LDH/CoP/CC	337	10	[48]
Ni_vac_Fe_vac_-LDH	240	100	[49]
FeNi LDH/Ti_3_C_2_-Mxene	298	50	[50]
Fe^2+^-NiFe-LDH	260	20	[51]
NiFe LDHs-VNi	184	20	[52]
PM-NiFe-LDH	230	100	[53]
NiFe	189	100	[54]
NiFeO_x_H_y_-PN	265	100	[55]
NiFe LDH nanosheets	260	42	[56]
NiFe-hollow nanoprisms	280	35	[57]
NiCoFe-NDA/NF	215	100	[58]
FeOOH/NiFe-LDH	265	200	[59]
d-NiFe-LDH	170	900	[37]
FeCoNi-LDHs	269	100	[60]
NiVIr-LDH	180	400	[61]
G-FeCoW	315	550	[62]
HE-LDHs-V+/CC	227	200	[63]
Au_SA_-MnFeCoNiCuLDH	213	700	[64]
O_2_-cat-1 (NiFe-LDH)	269	5000	[65]
CWO-del-48	288	608	[66]

## Data Availability

Data are contained within the article.
